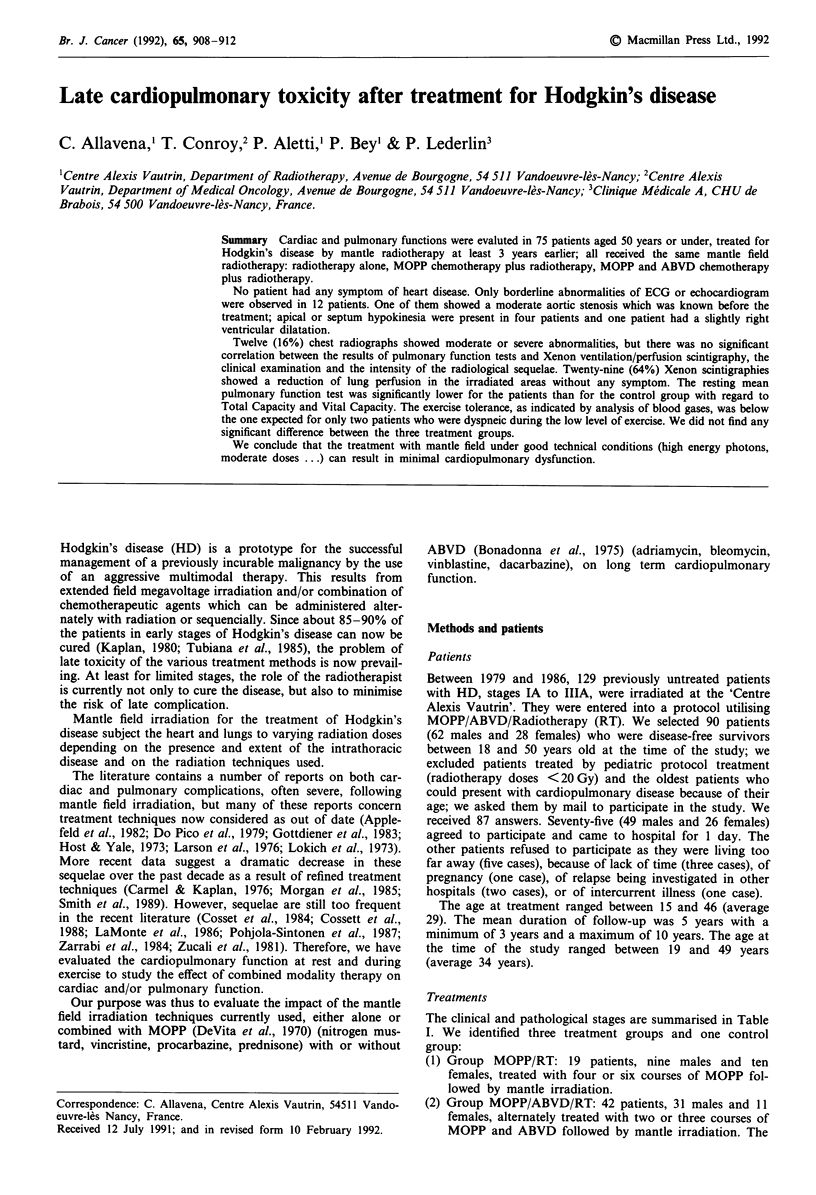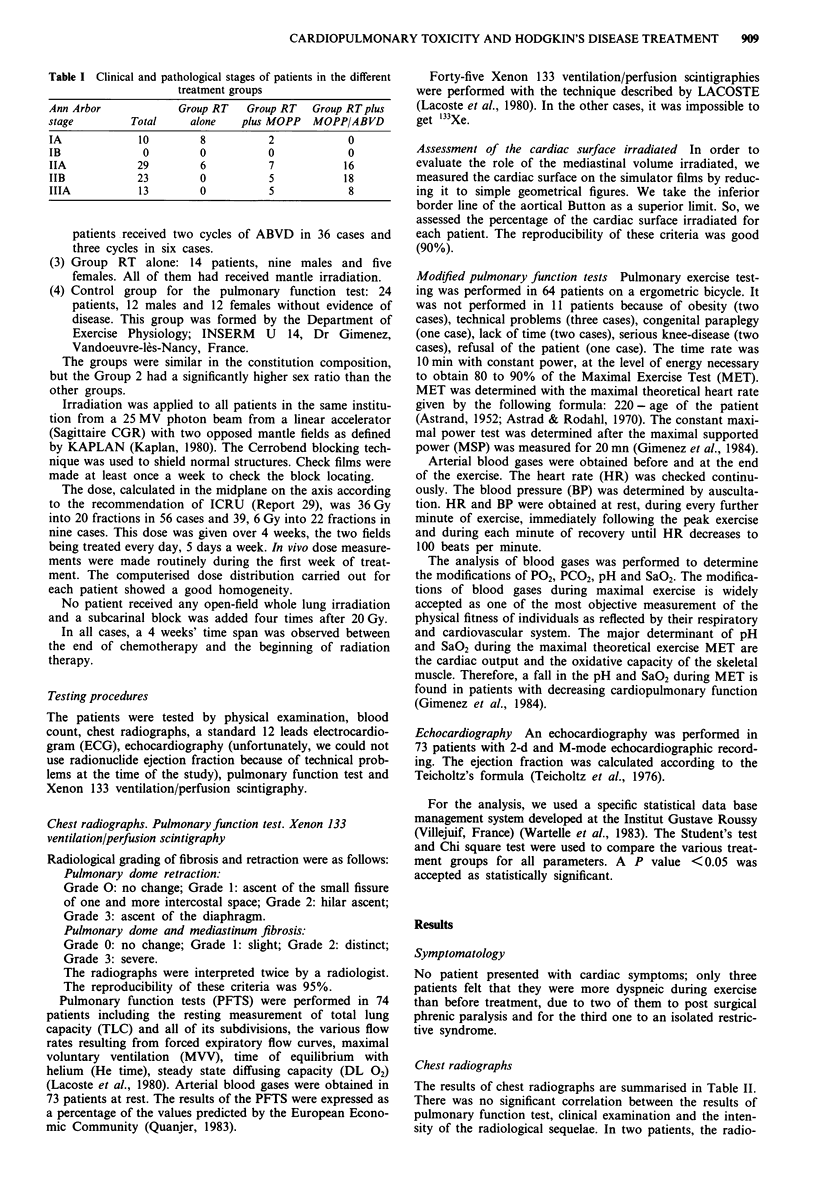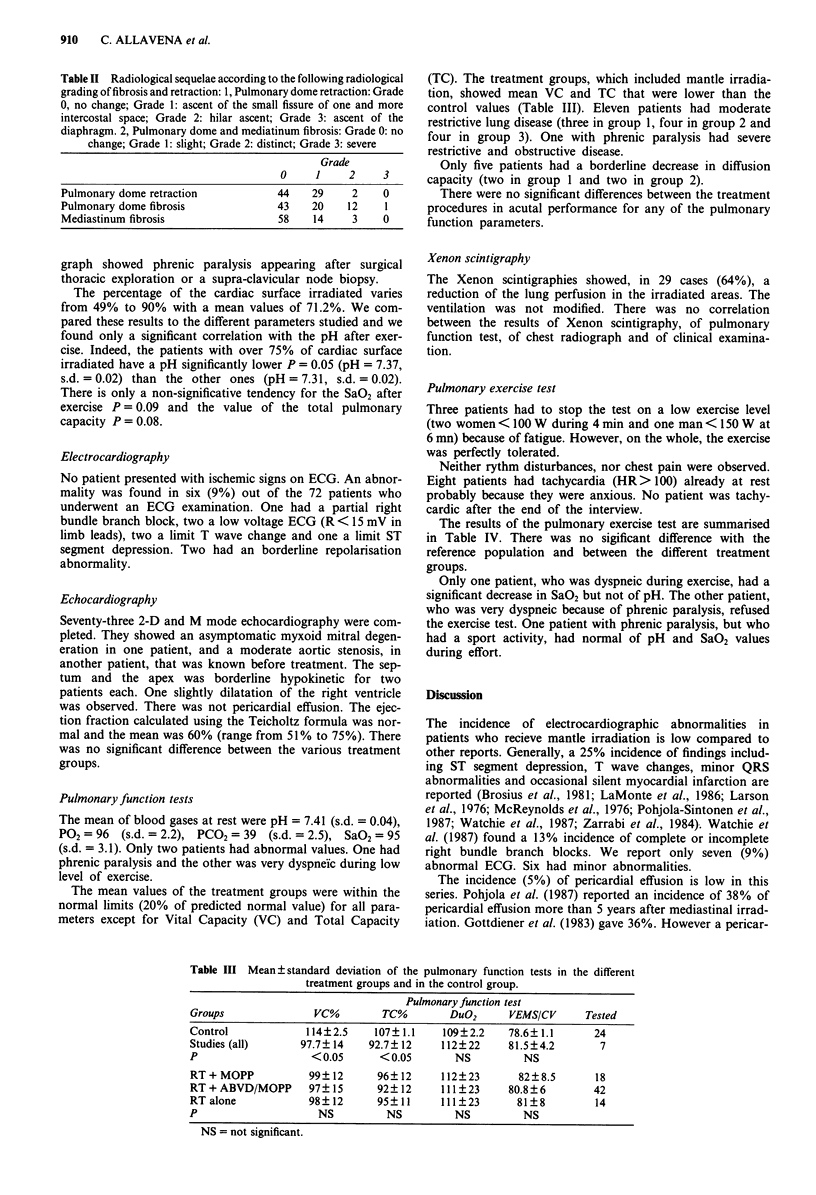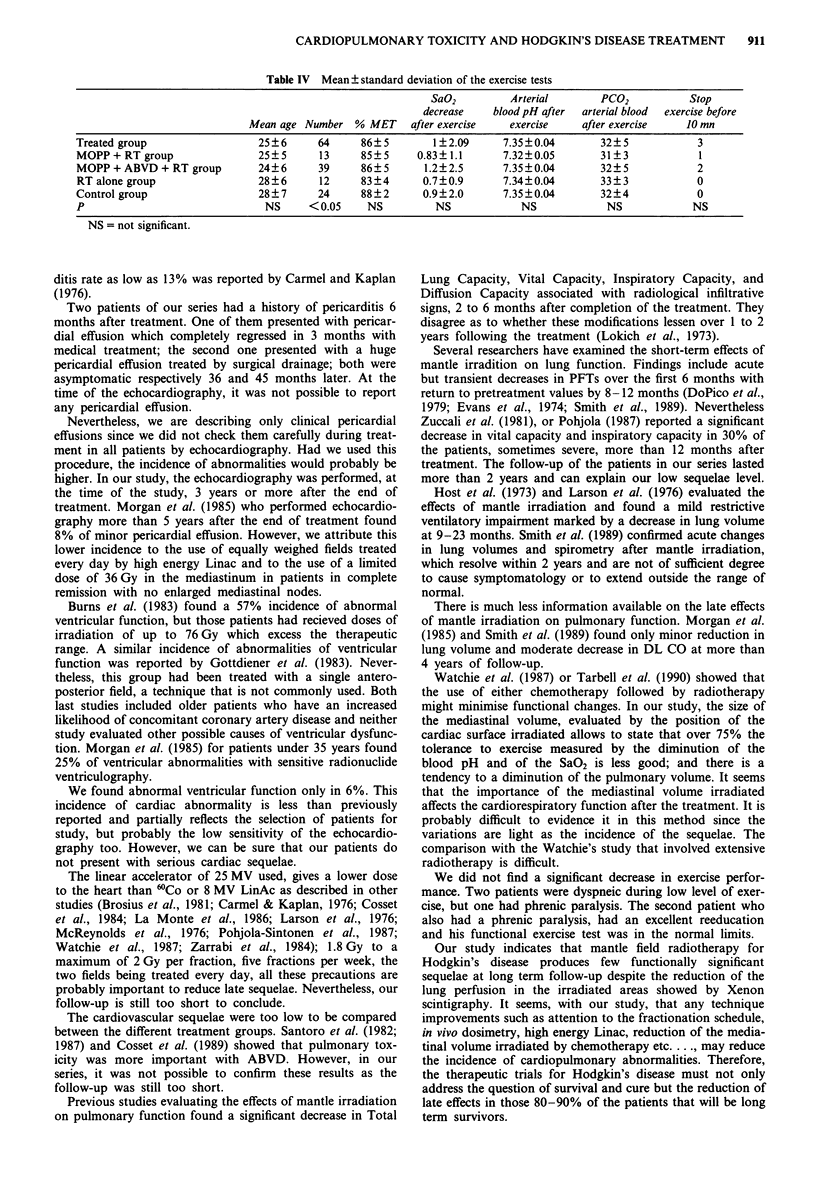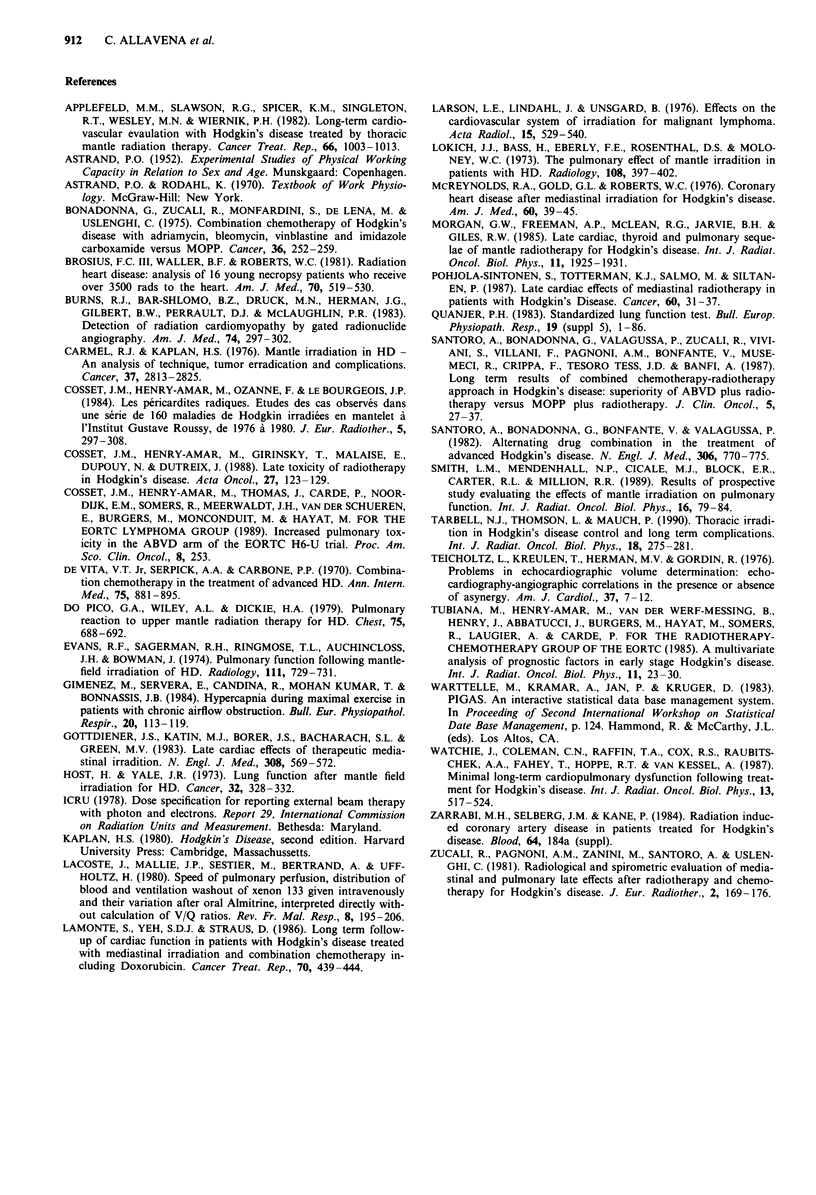# Late cardiopulmonary toxicity after treatment for Hodgkin's disease.

**DOI:** 10.1038/bjc.1992.190

**Published:** 1992-06

**Authors:** C. Allavena, T. Conroy, P. Aletti, P. Bey, P. Lederlin

**Affiliations:** Centre Alexis Vautrin, Department of Radiotherapy, Vandoeuvre-lès-Nancy, France.

## Abstract

Cardiac and pulmonary functions were evaluated in 75 patients aged 50 years or under, treated for Hodgkin's disease by mantle radiotherapy at least 3 years earlier; all received the same mantle field radiotherapy: radiotherapy alone, MOPP chemotherapy plus radiotherapy, MOPP and ABVD chemotherapy plus radiotherapy. No patient had any symptom of heart disease. Only borderline abnormalities of ECG or echocardiogram were observed in 12 patients. One of them showed a moderate aortic stenosis which was known before the treatment; apical or septum hypokinesia were present in four patients and one patient had a slightly right ventricular dilatation. Twelve (16%) chest radiographs showed moderate or severe abnormalities, but there was no significant correlation between the results of pulmonary function tests and Xenon ventilation/perfusion scintigraphy, the clinical examination and the intensity of the radiological sequelae. Twenty-nine (64%) Xenon scintigraphies showed a reduction of lung perfusion in the irradiated areas without any symptom. The resting mean pulmonary function test was significantly lower for the patients than for the control group with regard to Total Capacity and Vital Capacity. The exercise tolerance, as indicated by analysis of blood gases, was below the one expected for only two patients who were dyspneic during the low level of exercise. We did not find any significant difference between the three treatment groups. We conclude that the treatment with mantle field under good technical conditions (high energy photons, moderate doses...) can result in minimal cardiopulmonary dysfunction.


 © Macmillan Press Ltd., 1992

### Late cardiopulmonary toxicity after treatment for Hodgkin's disease

C. Allavena,[1] T. Conroy,[2] P. Aletti,[1] P. Bey[1] & P. Lederlin[3]

[1]*Centre Alexis Vautrin, Department of Radiotherapy, Avenue de Bourgogne, 54 511 Vandoeuvre-lès-Nancy;* [2]*Centre Alexis Vautrin, Department of Medical Oncology, Avenue de Bourgogne, 54 511 Vandoeuvre-lès-Nancy;* [3]*Clinique Médicale A, CHU de Brabois, 54 500 Vandoeuvre-lès-Nancy, France.*

**Summary** Cardiac and pulmonary functions were evaluted in 75 patients aged 50 years or under, treated for Hodgkin's disease by mantle radiotherapy at least 3 years earlier; all received the same mantle field radiotherapy: radiotherapy alone, MOPP chemotherapy plus radiotherapy, MOPP and ABVD chemotherapy plus radiotherapy.

No patient had any symptom of heart disease. Only borderline abnormalities of ECG or echocardiogram were observed in 12 patients. One of them showed a moderate aortic stenosis which was known before the treatment; apical or septum hypokinesia were present in four patients and one patient had a slightly right ventricular dilatation.

Twelve (16%) chest radiographs showed moderate or severe abnormalities, but there was no significant correlation between the results of pulmonary function tests and Xenon ventilation/perfusion scintigraphy, the clinical examination and the intensity of the radiological sequelae. Twenty-nine (64%) Xenon scintigraphies showed a reduction of lung perfusion in the irradiated areas without any symptom. The resting mean pulmonary function test was significantly lower for the patients than for the control group with regard to Total Capacity and Vital Capacity. The exercise tolerance, as indicated by analysis of blood gases, was below the one expected for only two patients who were dyspneic during the low level of exercise. We did not find any significant difference between the three treatment groups.

We conclude that the treatment with mantle field under good technical conditions (high energy photons, moderate doses ...) can result in minimal cardiopulmonary dysfunction.

Hodgkin's disease (HD) is a prototype for the successful management of a previously incurable malignancy by the use of an aggressive multimodal therapy. This results from extended field megavoltage irradiation and/or combination of chemotherapeutic agents which can be administered alternately with radiation or sequencially. Since about 85–90% of the patients in early stages of Hodgkin's disease can now be cured (Kaplan, 1980; Tubiana *et al.*, 1985), the problem of late toxicity of the various treatment methods is now prevailing. At least for limited stages, the role of the radiotherapist is currently not only to cure the disease, but also to minimise the risk of late complication.

Mantle field irradiation for the treatment of Hodgkin's disease subject the heart and lungs to varying radiation doses depending on the presence and extent of the intrathoracic disease and on the radiation techniques used.

The literature contains a number of reports on both cardiac and pulmonary complications, often severe, following mantle field irradiation, but many of these reports concern treatment techniques now considered as out of date (Applefeld *et al.*, 1982; Do Pico *et al.*, 1979; Gottdiener *et al.*, 1983; Host & Yale, 1973; Larson *et al.*, 1976; Lokich *et al.*, 1973). More recent data suggest a dramatic decrease in these sequelae over the past decade as a result of refined treatment techniques (Carmel & Kaplan, 1976; Morgan *et al.*, 1985; Smith *et al.*, 1989). However, sequelae are still too frequent in the recent literature (Cosset *et al.*, 1984; Cossett *et al.*, 1988; LaMonte *et al.*, 1986; Pohjola-Sintonen *et al.*, 1987; Zarrabi *et al.*, 1984; Zucali *et al.*, 1981). Therefore, we have evaluated the cardiopulmonary function at rest and during exercise to study the effect of combined modality therapy on cardiac and/or pulmonary function.

Our purpose was thus to evaluate the impact of the mantle field irradiation techniques currently used, either alone or combined with MOPP (DeVita *et al.*, 1970) (nitrogen mustard, vincristine, procarbazine, prednisone) with or without ABVD (Bonadonna *et al.*, 1975) (adriamycin, bleomycin, vinblastine, dacarbazine), on long term cardiopulmonary function.

Correspondence: C. Allavena, Centre Alexis Vautrin, 54511 Vandoeuvre-lès Nancy, France.
Received 12 July 1991; and in revised form 10 February 1992.

### Methods and patients

### *Patients*

Between 1979 and 1986, 129 previously untreated patients with HD, stages IA to IIIA, were irradiated at the 'Centre Alexis Vautrin'. They were entered into a protocol utilising MOPP/ABVD/Radiotherapy (RT). We selected 90 patients (62 males and 28 females) who were disease-free survivors between 18 and 50 years old at the time of the study; we excluded patients treated by pediatric protocol treatment (radiotherapy doses <20 Gy) and the oldest patients who could present with cardiopulmonary disease because of their age; we asked them by mail to participate in the study. We received 87 answers. Seventy-five (49 males and 26 females) agreed to participate and came to hospital for 1 day. The other patients refused to participate as they were living too far away (five cases), because of lack of time (three cases), of pregnancy (one case), of relapse being investigated in other hospitals (two cases), or of intercurrent illness (one case).

The age at treatment ranged between 15 and 46 (average 29). The mean duration of follow-up was 5 years with a minimum of 3 years and a maximum of 10 years. The age at the time of the study ranged between 19 and 49 years (average 34 years).

### *Treatments*

The clinical and pathological stages are summarised in Table I. We identified three treatment groups and one control group:

(1) Group MOPP/RT: 19 patients, nine males and ten females, treated with four or six courses of MOPP followed by mantle irradiation.
(2) Group MOPP/ABVD/RT: 42 patients, 31 males and 11 females, alternately treated with two or three courses of MOPP and ABVD followed by mantle irradiation. The

**Table I** Clinical and pathological stages of patients in the different treatment groups

| *Ann Arbor stage* | *Total* | *Group RT alone* | *Group RT plus MOPP* | *Group RT plus MOPP/ABVD* |
|---|---|---|---|---|
| IA | 10 | 8 | 2 | 0 |
| IB | 0 | 0 | 0 | 0 |
| IIA | 29 | 6 | 7 | 16 |
| IIB | 23 | 0 | 5 | 18 |
| IIIA | 13 | 0 | 5 | 8 |

patients received two cycles of ABVD in 36 cases and three cycles in six cases.

(3) Group RT alone: 14 patients, nine males and five females. All of them had received mantle irradiation.

(4) Control group for the pulmonary function test: 24 patients, 12 males and 12 females without evidence of disease. This group was formed by the Department of Exercise Physiology; INSERM U 14, Dr Gimenez, Vandoeuvre-lès-Nancy, France.

The groups were similar in the constitution composition, but the Group 2 had a significantly higher sex ratio than the other groups.

Irradiation was applied to all patients in the same institution from a 25 MV photon beam from a linear accelerator (Sagittaire CGR) with two opposed mantle fields as defined by KAPLAN (Kaplan, 1980). The Cerrobend blocking technique was used to shield normal structures. Check films were made at least once a week to check the block locating.

The dose, calculated in the midplane on the axis according to the recommendation of ICRU (Report 29), was 36 Gy into 20 fractions in 56 cases and 39, 6 Gy into 22 fractions in nine cases. This dose was given over 4 weeks, the two fields being treated every day, 5 days a week. *In vivo* dose measurements were made routinely during the first week of treatment. The computerised dose distribution carried out for each patient showed a good homogeneity.

No patient received any open-field whole lung irradiation and a subcarinal block was added four times after 20 Gy.

In all cases, a 4 weeks' time span was observed between the end of chemotherapy and the beginning of radiation therapy.

### *Testing procedures*

The patients were tested by physical examination, blood count, chest radiographs, a standard 12 leads electrocardiogram (ECG), echocardiography (unfortunately, we could not use radionuclide ejection fraction because of technical problems at the time of the study), pulmonary function test and Xenon 133 ventilation/perfusion scintigraphy.

### *Chest radiographs. Pulmonary function test. Xenon 133 ventilation/perfusion scintigraphy*

Radiological grading of fibrosis and retraction were as follows:

*Pulmonary dome retraction:*

Grade O: no change; Grade 1: ascent of the small fissure of one and more intercostal space; Grade 2: hilar ascent; Grade 3: ascent of the diaphragm.

*Pulmonary dome and mediastinum fibrosis:*

Grade 0: no change; Grade 1: slight; Grade 2: distinct; Grade 3: severe.

The radiographs were interpreted twice by a radiologist. The reproducibility of these criteria was 95%.

Pulmonary function tests (PFTS) were performed in 74 patients including the resting measurement of total lung capacity (TLC) and all of its subdivisions, the various flow rates resulting from forced expiratory flow curves, maximal voluntary ventilation (MVV), time of equilibrium with helium (He time), steady state diffusing capacity (DL $O_2$) (Lacoste *et al.*, 1980). Arterial blood gases were obtained in 73 patients at rest. The results of the PFTS were expressed as a percentage of the values predicted by the European Economic Community (Quanjer, 1983).

Forty-five Xenon 133 ventilation/perfusion scintigraphies were performed with the technique described by LACOSTE (Lacoste *et al.*, 1980). In the other cases, it was impossible to get $^{133}$Xe.

*Assessment of the cardiac surface irradiated* In order to evaluate the role of the mediastinal volume irradiated, we measured the cardiac surface on the simulator films by reducing it to simple geometrical figures. We take the inferior border line of the aortical Button as a superior limit. So, we assessed the percentage of the cardiac surface irradiated for each patient. The reproducibility of these criteria was good (90%).

*Modified pulmonary function tests* Pulmonary exercise testing was performed in 64 patients on a ergometric bicycle. It was not performed in 11 patients because of obesity (two cases), technical problems (three cases), congenital paraplegy (one case), lack of time (two cases), serious knee-disease (two cases), refusal of the patient (one case). The time rate was 10 min with constant power, at the level of energy necessary to obtain 80 to 90% of the Maximal Exercise Test (MET). MET was determined with the maximal theoretical heart rate given by the following formula: 220 − age of the patient (Astrand, 1952; Astrad & Rodahl, 1970). The constant maximal power test was determined after the maximal supported power (MSP) was measured for 20 mn (Gimenez *et al.*, 1984).

Arterial blood gases were obtained before and at the end of the exercise. The heart rate (HR) was checked continuously. The blood pressure (BP) was determined by auscultation. HR and BP were obtained at rest, during every further minute of exercise, immediately following the peak exercise and during each minute of recovery until HR decreases to 100 beats per minute.

The analysis of blood gases was performed to determine the modifications of $PO_2$, $PCO_2$, pH and $SaO_2$. The modifications of blood gases during maximal exercise is widely accepted as one of the most objective measurement of the physical fitness of individuals as reflected by their respiratory and cardiovascular system. The major determinant of pH and $SaO_2$ during the maximal theoretical exercise MET are the cardiac output and the oxidative capacity of the skeletal muscle. Therefore, a fall in the pH and $SaO_2$ during MET is found in patients with decreasing cardiopulmonary function (Gimenez *et al.*, 1984).

*Echocardiography* An echocardiography was performed in 73 patients with 2-d and M-mode echocardiographic recording. The ejection fraction was calculated according to the Teicholtz's formula (Teicholtz *et al.*, 1976).

For the analysis, we used a specific statistical data base management system developed at the Institut Gustave Roussy (Villejuif, France) (Wartelle *et al.*, 1983). The Student's test and Chi square test were used to compare the various treatment groups for all parameters. A $P$ value $<0.05$ was accepted as statistically significant.

### Results

### *Symptomatology*

No patient presented with cardiac symptoms; only three patients felt that they were more dyspneic during exercise than before treatment, due to two of them to post surgical phrenic paralysis and for the third one to an isolated restrictive syndrome.

### *Chest radiographs*

The results of chest radiographs are summarised in Table II. There was no significant correlation between the results of pulmonary function test, clinical examination and the intensity of the radiological sequelae. In two patients, the radio-

**Table II** Radiological sequelae according to the following radiological grading of fibrosis and retraction: 1, Pulmonary dome retraction: Grade 0, no change; Grade 1: ascent of the small fissure of one and more intercostal space; Grade 2: hilar ascent; Grade 3: ascent of the diaphragm. 2, Pulmonary dome and mediatinum fibrosis: Grade 0: no change; Grade 1: slight; Grade 2: distinct; Grade 3: severe

| | *Grade* 0 | 1 | 2 | 3 |
|---|---|---|---|---|
| Pulmonary dome retraction | 44 | 29 | 2 | 0 |
| Pulmonary dome fibrosis | 43 | 20 | 12 | 1 |
| Mediastinum fibrosis | 58 | 14 | 3 | 0 |

graph showed phrenic paralysis appearing after surgical thoracic exploration or a supra-clavicular node biopsy.

The percentage of the cardiac surface irradiated varies from 49% to 90% with a mean values of 71.2%. We compared these results to the different parameters studied and we found only a significant correlation with the pH after exercise. Indeed, the patients with over 75% of cardiac surface irradiated have a pH significantly lower $P = 0.05$ (pH = 7.37, s.d. = 0.02) than the other ones (pH = 7.31, s.d. = 0.02). There is only a non-significative tendency for the $SaO_2$ after exercise $P = 0.09$ and the value of the total pulmonary capacity $P = 0.08$.

### *Electrocardiography*

No patient presented with ischemic signs on ECG. An abnormality was found in six (9%) out of the 72 patients who underwent an ECG examination. One had a partial right bundle branch block, two a low voltage ECG (R < 15 mV in limb leads), two a limit T wave change and one a limit ST segment depression. Two had an borderline repolarisation abnormality.

### *Echocardiography*

Seventy-three 2-D and M mode echocardiography were completed. They showed an asymptomatic myxoid mitral degeneration in one patient, and a moderate aortic stenosis, in another patient, that was known before treatment. The septum and the apex was borderline hypokinetic for two patients each. One slightly dilatation of the right ventricle was observed. There was not pericardial effusion. The ejection fraction calculated using the Teicholtz formula was normal and the mean was 60% (range from 51% to 75%). There was no significant difference between the various treatment groups.

### *Pulmonary function tests*

The mean of blood gases at rest were pH = 7.41 (s.d. = 0.04), $PO_2 = 96$ (s.d. = 2.2), $PCO_2 = 39$ (s.d. = 2.5), $SaO_2 = 95$ (s.d. = 3.1). Only two patients had abnormal values. One had phrenic paralysis and the other was very dyspneïc during low level of exercise.

The mean values of the treatment groups were within the normal limits (20% of predicted normal value) for all parameters except for Vital Capacity (VC) and Total Capacity (TC). The treatment groups, which included mantle irradiation, showed mean VC and TC that were lower than the control values (Table III). Eleven patients had moderate restrictive lung disease (three in group 1, four in group 2 and four in group 3). One with phrenic paralysis had severe restrictive and obstructive disease.

Only five patients had a borderline decrease in diffusion capacity (two in group 1 and two in group 2).

There were no significant differences between the treatment procedures in acutal performance for any of the pulmonary function parameters.

### *Xenon scintigraphy*

The Xenon scintigraphies showed, in 29 cases (64%), a reduction of the lung perfusion in the irradiated areas. The ventilation was not modified. There was no correlation between the results of Xenon scintigraphy, of pulmonary function test, of chest radiograph and of clinical examination.

### *Pulmonary exercise test*

Three patients had to stop the test on a low exercise level (two women < 100 W during 4 min and one man < 150 W at 6 mn) because of fatigue. However, on the whole, the exercise was perfectly tolerated.

Neither rythm disturbances, nor chest pain were observed. Eight patients had tachycardia (HR > 100) already at rest probably because they were anxious. No patient was tachycardic after the end of the interview.

The results of the pulmonary exercise test are summarised in Table IV. There was no sigificant difference with the reference population and between the different treatment groups.

Only one patient, who was dyspneic during exercise, had a significant decrease in $SaO_2$ but not of pH. The other patient, who was very dyspneic because of phrenic paralysis, refused the exercise test. One patient with phrenic paralysis, but who had a sport activity, had normal of pH and $SaO_2$ values during effort.

### Discussion

The incidence of electrocardiographic abnormalities in patients who recieve mantle irradiation is low compared to other reports. Generally, a 25% incidence of findings including ST segment depression, T wave changes, minor QRS abnormalities and occasional silent myocardial infarction are reported (Brosius *et al.*, 1981; LaMonte *et al.*, 1986; Larson *et al.*, 1976; McReynolds *et al.*, 1976; Pohjola-Sintonen *et al.*, 1987; Watchie *et al.*, 1987; Zarrabi *et al.*, 1984). Watchie *et al.* (1987) found a 13% incidence of complete or incomplete right bundle branch blocks. We report only seven (9%) abnormal ECG. Six had minor abnormalities.

The incidence (5%) of pericardial effusion is low in this series. Pohjola *et al.* (1987) reported an incidence of 38% of pericardial effusion more than 5 years after mediastinal irradiation. Gottdiener *et al.* (1983) gave 36%. However a pericar-

**Table III** Mean ± standard deviation of the pulmonary function tests in the different treatment groups and in the control group.

| *Groups* | *Pulmonary function test* *VC%* | *TC%* | *$DuO_2$* | *VEMS/CV* | *Tested* |
|---|---|---|---|---|---|
| Control | 114±2.5 | 107±1.1 | 109±2.2 | 78.6±1.1 | 24 |
| Studies (all) | 97.7±14 | 92.7±12 | 112±22 | 81.5±4.2 | 7 |
| *P* | <0.05 | <0.05 | NS | NS | |
| RT + MOPP | 99±12 | 96±12 | 112±23 | 82±8.5 | 18 |
| RT + ABVD/MOPP | 97±15 | 92±12 | 111±23 | 80.8±6 | 42 |
| RT alone | 98±12 | 95±11 | 111±23 | 81±8 | 14 |
| *P* | NS | NS | NS | NS | |

NS = not significant.

**Table IV** Mean ± standard deviation of the exercise tests

| | *Mean age* | *Number* | *% MET* | *$SaO_2$ decrease after exercise* | *Arterial blood pH after exercise* | *$PCO_2$ arterial blood after exercise* | *Stop exercise before 10 mn* |
|---|---|---|---|---|---|---|---|
| Treated group | 25±6 | 64 | 86±5 | 1±2.09 | 7.35±0.04 | 32±5 | 3 |
| MOPP + RT group | 25±5 | 13 | 85±5 | 0.83±1.1 | 7.32±0.05 | 31±3 | 1 |
| MOPP + ABVD + RT group | 24±6 | 39 | 86±5 | 1.2±2.5 | 7.35±0.04 | 32±5 | 2 |
| RT alone group | 28±6 | 12 | 83±4 | 0.7±0.9 | 7.34±0.04 | 33±3 | 0 |
| Control group | 28±7 | 24 | 88±2 | 0.9±2.0 | 7.35±0.04 | 32±4 | 0 |
| *P* | NS | <0.05 | NS | NS | NS | NS | NS |

NS = not significant.

ditis rate as low as 13% was reported by Carmel and Kaplan (1976).

Two patients of our series had a history of pericarditis 6 months after treatment. One of them presented with pericardial effusion which completely regressed in 3 months with medical treatment; the second one presented with a huge pericardial effusion treated by surgical drainage; both were asymptomatic respectively 36 and 45 months later. At the time of the echocardiography, it was not possible to report any pericardial effusion.

Nevertheless, we are describing only clinical pericardial effusions since we did not check them carefully during treatment in all patients by echocardiography. Had we used this procedure, the incidence of abnormalities would probably be higher. In our study, the echocardiography was performed, at the time of the study, 3 years or more after the end of treatment. Morgan *et al.* (1985) who performed echocardiography more than 5 years after the end of treatment found 8% of minor pericardial effusion. However, we attribute this lower incidence to the use of equally weighed fields treated every day by high energy Linac and to the use of a limited dose of 36 Gy in the mediastinum in patients in complete remission with no enlarged mediastinal nodes.

Burns *et al.* (1983) found a 57% incidence of abnormal ventricular function, but those patients had recieved doses of irradiation of up to 76 Gy which excess the therapeutic range. A similar incidence of abnormalities of ventricular function was reported by Gottdiener *et al.* (1983). Nevertheless, this group had been treated with a single antero-posterior field, a technique that is not commonly used. Both last studies included older patients who have an increased likelihood of concomitant coronary artery disease and neither study evaluated other possible causes of ventricular dysfunction. Morgan *et al.* (1985) for patients under 35 years found 25% of ventricular abnormalities with sensitive radionuclide ventriculography.

We found abnormal ventricular function only in 6%. This incidence of cardiac abnormality is less than previously reported and partially reflects the selection of patients for study, but probably the low sensitivity of the echocardiography too. However, we can be sure that our patients do not present with serious cardiac sequelae.

The linear accelerator of 25 MV used, gives a lower dose to the heart than $^{60}Co$ or 8 MV LinAc as described in other studies (Brosius *et al.*, 1981; Carmel & Kaplan, 1976; Cosset *et al.*, 1984; La Monte *et al.*, 1986; Larson *et al.*, 1976; McReynolds *et al.*, 1976; Pohjola-Sintonen *et al.*, 1987; Watchie *et al.*, 1987; Zarrabi *et al.*, 1984); 1.8 Gy to a maximum of 2 Gy per fraction, five fractions per week, the two fields being treated every day, all these precautions are probably important to reduce late sequelae. Nevertheless, our follow-up is still too short to conclude.

The cardiovascular sequelae were too low to be compared between the different treatment groups. Santoro *et al.* (1982; 1987) and Cosset *et al.* (1989) showed that pulmonary toxicity was more important with ABVD. However, in our series, it was not possible to confirm these results as the follow-up was still too short.

Previous studies evaluating the effects of mantle irradiation on pulmonary function found a significant decrease in Total Lung Capacity, Vital Capacity, Inspiratory Capacity, and Diffusion Capacity associated with radiological infiltrative signs, 2 to 6 months after completion of the treatment. They disagree as to whether these modifications lessen over 1 to 2 years following the treatment (Lokich *et al.*, 1973).

Several researchers have examined the short-term effects of mantle irradition on lung function. Findings include acute but transient decreases in PFTs over the first 6 months with return to pretreatment values by 8–12 months (DoPico *et al.*, 1979; Evans *et al.*, 1974; Smith *et al.*, 1989). Nevertheless Zuccali *et al.* (1981), or Pohjola (1987) reported a significant decrease in vital capacity and inspiratory capacity in 30% of the patients, sometimes severe, more than 12 months after treatment. The follow-up of the patients in our series lasted more than 2 years and can explain our low sequelae level.

Host *et al.* (1973) and Larson *et al.* (1976) evaluated the effects of mantle irradiation and found a mild restrictive ventilatory impairment marked by a decrease in lung volume at 9–23 months. Smith *et al.* (1989) confirmed acute changes in lung volumes and spirometry after mantle irradiation, which resolve within 2 years and are not of sufficient degree to cause symptomatology or to extend outside the range of normal.

There is much less information available on the late effects of mantle irradiation on pulmonary function. Morgan *et al.* (1985) and Smith *et al.* (1989) found only minor reduction in lung volume and moderate decrease in DL CO at more than 4 years of follow-up.

Watchie *et al.* (1987) or Tarbell *et al.* (1990) showed that the use of either chemotherapy followed by radiotherapy might minimise functional changes. In our study, the size of the mediastinal volume, evaluated by the position of the cardiac surface irradiated allows to state that over 75% the tolerance to exercise measured by the diminution of the blood pH and of the $SaO_2$ is less good; and there is a tendency to a diminution of the pulmonary volume. It seems that the importance of the mediastinal volume irradiated affects the cardiorespiratory function after the treatment. It is probably difficult to evidence it in this method since the variations are light as the incidence of the sequelae. The comparison with the Watchie's study that involved extensive radiotherapy is difficult.

We did not find a significant decrease in exercise performance. Two patients were dyspneic during low level of exercise, but one had phrenic paralysis. The second patient who also had a phrenic paralysis, had an excellent reeducation and his functional exercise test was in the normal limits.

Our study indicates that mantle field radiotherapy for Hodgkin's disease produces few functionally significant sequelae at long term follow-up despite the reduction of the lung perfusion in the irradiated areas showed by Xenon scintigraphy. It seems, with our study, that any technique improvements such as attention to the fractionation schedule, *in vivo* dosimetry, high energy Linac, reduction of the mediatinal volume irradiated by chemotherapy etc. . . ., may reduce the incidence of cardiopulmonary abnormalities. Therefore, the therapeutic trials for Hodgkin's disease must not only address the question of survival and cure but the reduction of late effects in those 80–90% of the patients that will be long term survivors.

### References

APPLEFELD, M.M., SLAWSON, R.G., SPICER, K.M., SINGLETON, R.T., WESLEY, M.N. & WIERNIK, P.H. (1982). Long-term cardiovascular evaulation with Hodgkin's disease treated by thoracic mantle radiation therapy. *Cancer Treat. Rep.*, **66**, 1003–1013.

ASTRAND, P.O. (1952). *Experimental Studies of Physical Working Capacity in Relation to Sex and Age*. Munskgaard: Copenhagen.

ASTRAND, P.O. & RODAHL, K. (1970). *Textbook of Work Physiology*. McGraw-Hill: New York.

BONADONNA, G., ZUCALI, R., MONFARDINI, S., DE LENA, M. & USLENGHI, C. (1975). Combination chemotherapy of Hodgkin's disease with adriamycin, bleomycin, vinblastine and imidazole carboxamide versus MOPP. *Cancer*, **36**, 252–259.

BROSIUS, F.C. III, WALLER, B.F. & ROBERTS, W.C. (1981). Radiation heart disease: analysis of 16 young necropsy patients who receive over 3500 rads to the heart. *Am. J. Med.*, **70**, 519–530.

BURNS, R.J., BAR-SHLOMO, B.Z., DRUCK, M.N., HERMAN, J.G., GILBERT, B.W., PERRAULT, D.J. & MCLAUGHLIN, P.R. (1983). Detection of radiation cardiomyopathy by gated radionuclide angiography. *Am. J. Med.*, **74**, 297–302.

CARMEL, R.J. & KAPLAN, H.S. (1976). Mantle irradiation in HD – An analysis of technique, tumor erradication and complications. *Cancer*, **37**, 2813–2825.

COSSET, J.M., HENRY-AMAR, M., OZANNE, F. & LE BOURGEOIS, J.P. (1984). Les péricardites radiques. Etudes des cas observés dans une série de 160 maladies de Hodgkin irradiées en mantelet à l'Institut Gustave Roussy, de 1976 à 1980. *J. Eur. Radiother.*, **5**, 297–308.

COSSET, J.M., HENRY-AMAR, M., GIRINSKY, T., MALAISE, E., DUPOUY, N. & DUTREIX, J. (1988). Late toxicity of radiotherapy in Hodgkin's disease. *Acta Oncol.*, **27**, 123–129.

COSSET, J.M., HENRY-AMAR, M., THOMAS, J., CARDE, P., NOORDIJK, E.M., SOMERS, R., MEERWALDT, J.H., VAN DER SCHUEREN, E., BURGERS, M., MONCONDUIT, M. & HAYAT, M. FOR THE EORTC LYMPHOMA GROUP (1989). Increased pulmonary toxicity in the ABVD arm of the EORTC H6-U trial. *Proc. Am. Sco. Clin. Oncol.*, **8**, 253.

DE VITA, V.T. Jr, SERPICK, A.A. & CARBONE, P.P. (1970). Combination chemotherapy in the treatment of advanced HD. *Ann. Intern. Med.*, **75**, 881–895.

DO PICO, G.A., WILEY, A.L. & DICKIE, H.A. (1979). Pulmonary reaction to upper mantle radiation therapy for HD. *Chest*, **75**, 688–692.

EVANS, R.F., SAGERMAN, R.H., RINGMOSE, T.L., AUCHINCLOSS, J.H. & BOWMAN, J. (1974). Pulmonary function following mantle-field irradiation of HD. *Radiology*, **111**, 729–731.

GIMENEZ, M., SERVERA, E., CANDINA, R., MOHAN KUMAR, T. & BONNASSIS, J.B. (1984). Hypercapnia during maximal exercise in patients with chronic airflow obstruction. *Bull. Eur. Physiopathol. Respir.*, **20**, 113–119.

GOTTDIENER, J.S., KATIN, M.J., BORER, J.S., BACHARACH, S.L. & GREEN, M.V. (1983). Late cardiac effects of therapeutic mediastinal irradition. *N. Engl. J. Med.*, **308**, 569–572.

HOST, H. & YALE, J.R. (1973). Lung function after mantle field irradiation for HD. *Cancer*, **32**, 328–332.

ICRU (1978). Dose specification for reporting external beam therapy with photon and electrons. *Report 29, International Commission on Radiation Units and Measurement*. Bethesda: Maryland.

KAPLAN, H.S. (1980). *Hodgkin's Disease*, second edition. Harvard University Press: Cambridge, Massachussetts.

LACOSTE, J., MALLIE, J.P., SESTIER, M., BERTRAND, A. & UFFHOLTZ, H. (1980). Speed of pulmonary perfusion, distribution of blood and ventilation washout of xenon 133 given intravenously and their variation after oral Almitrine, interpreted directly without calculation of V/Q ratios. *Rev. Fr. Mal. Resp.*, **8**, 195–206.

LAMONTE, S., YEH, S.D.J. & STRAUS, D. (1986). Long term follow-up of cardiac function in patients with Hodgkin's disease treated with mediastinal irradiation and combination chemotherapy including Doxorubicin. *Cancer Treat. Rep.*, **70**, 439–444.

LARSON, L.E., LINDAHL, J. & UNSGARD, B. (1976). Effects on the cardiovascular system of irradiation for malignant lymphoma. *Acta Radiol.*, **15**, 529–540.

LOKICH, J.J., BASS, H., EBERLY, F.E., ROSENTHAL, D.S. & MOLONEY, W.C. (1973). The pulmonary effect of mantle irradition in patients with HD. *Radiology*, **108**, 397–402.

MCREYNOLDS, R.A., GOLD, G.L. & ROBERTS, W.C. (1976). Coronary heart disease after mediastinal irradiation for Hodgkin's disease. *Am. J. Med.*, **60**, 39–45.

MORGAN, G.W., FREEMAN, A.P., MCLEAN, R.G., JARVIE, B.H. & GILES, R.W. (1985). Late cardiac, thyroid and pulmonary sequelae of mantle radiotherapy for Hodgkin's disease. *Int. J. Radiat. Oncol. Biol. Phys.*, **11**, 1925–1931.

POHJOLA-SINTONEN, S., TOTTERMAN, K.J., SALMO, M. & SILTANEN, P. (1987). Late cardiac effects of mediastinal radiotherapy in patients with Hodgkin's Disease. *Cancer*, **60**, 31–37.

QUANJER, P.H. (1983). Standardized lung function test. *Bull. Europ. Physiopath. Resp.*, **19** (suppl 5), 1–86.

SANTORO, A., BONADONNA, G., VALAGUSSA, P., ZUCALI, R., VIVIANI, S., VILLANI, F., PAGNONI, A.M., BONFANTE, V., MUSEMECI, R., CRIPPA, F., TESORO TESS, J.D. & BANFI, A. (1987). Long term results of combined chemotherapy-radiotherapy approach in Hodgkin's disease: superiority of ABVD plus radiotherapy versus MOPP plus radiotherapy. *J. Clin. Oncol.*, **5**, 27–37.

SANTORO, A., BONADONNA, G., BONFANTE, V. & VALAGUSSA, P. (1982). Alternating drug combination in the treatment of advanced Hodgkin's disease. *N. Engl. J. Med.*, **306**, 770–775.

SMITH, L.M., MENDENHALL, N.P., CICALE, M.J., BLOCK, E.R., CARTER, R.L. & MILLION, R.R. (1989). Results of prospective study evaluating the effects of mantle irradiation on pulmonary function. *Int. J. Radiat. Oncol. Biol. Phys.*, **16**, 79–84.

TARBELL, N.J., THOMSON, L. & MAUCH, P. (1990). Thoracic irradition in Hodgkin's disease control and long term complications. *Int. J. Radiat. Oncol. Biol. Phys.*, **18**, 275–281.

TEICHOLTZ, L., KREULEN, T., HERMAN, M.V. & GORDIN, R. (1976). Problems in echocardiographic volume determination: echocardiography-angiographic correlations in the presence or absence of asynergy. *Am. J. Cardiol.*, **37**, 7–12.

TUBIANA, M., HENRY-AMAR, M., VAN DER WERF-MESSING, B., HENRY, J., ABBATUCCI, J., BURGERS, M., HAYAT, M., SOMERS, R., LAUGIER, A. & CARDE, P. FOR THE RADIOTHERAPY-CHEMOTHERAPY GROUP OF THE EORTC (1985). A multivariate analysis of prognostic factors in early stage Hodgkin's disease. *Int. J. Radiat. Oncol. Biol. Phys.*, **11**, 23–30.

WARTTELLE, M., KRAMAR, A., JAN, P. & KRUGER, D. (1983). PIGAS. An interactive statistical data base management system. In *Proceeding of Second International Workshop on Statistical Date Base Management*, p. 124. Hammond, R. & McCarthy, J.L. (eds). Los Altos, CA.

WATCHIE, J., COLEMAN, C.N., RAFFIN, T.A., COX, R.S., RAUBITSCHEK, A.A., FAHEY, T., HOPPE, R.T. & VAN KESSEL, A. (1987). Minimal long-term cardiopulmonary dysfunction following treatment for Hodgkin's disease. *Int. J. Radiat. Oncol. Biol. Phys.*, **13**, 517–524.

ZARRABI, M.H., SELBERG, J.M. & KANE, P. (1984). Radiation induced coronary artery disease in patients treated for Hodgkin's disease. *Blood*, **64**, 184a (suppl).

ZUCALI, R., PAGNONI, A.M., ZANINI, M., SANTORO, A. & USLENGHI, C. (1981). Radiological and spirometric evaluation of mediastinal and pulmonary late effects after radiotherapy and chemotherapy for Hodgkin's disease. *J. Eur. Radiother.*, **2**, 169–176.